# Evaluation of irrational drug use of individuals over the age of 18 who applied to a university hospital

**DOI:** 10.3906/sag-2110-215

**Published:** 2021-12-28

**Authors:** Sevil ÖZGER İLHAN, Mücahit YILDIZ, Hakan TÜZÜN, Asiye UĞRAŞ DİKMEN

**Affiliations:** 1 Department of Medical Pharmacology, Faculty of Medicine, Gazi University, Ankara, Turkey; 2 Department of Public Health, Faculty of Medicine, Gazi University, Ankara, Turkey

**Keywords:** Irrational drug use, self-medication, health determinants, cross-sectional study

## Abstract

**Background/aim:**

Irrational drug use not only causes a delay in the treatment of patients, failure to achieve full well-being, drug interactions and side effects, drug resistance but also creates economic negativities such as waste of resources and unnecessary workload. This study aims to investigate the irrational drug use behaviors of individuals over the age of 18 who applied to a university hospital.

**Materials and methods:**

This sectional study included 1247 people over the age of 18 who applied for Gazi University Health, Research and Application Center. A questionnaire was applied to the applicants by face-to-face interview technique. Logistic regression analysis was performed among the factors associated with the subcomponents of irrational drug use.

**Results:**

Participants (20.1%) used medication without a doctor’s prescription (self-medication), 3.4% did not comply with the recommended dose and duration for medications, 47.4% applied to the physician to prescribe the medication they wanted, 65% had medication for later use at home. It was determined that 24.1% of them used drugs without looking at the expiration date and 45.5% of them used drugs without reading the patient information leaflet. Among the risk groups identified for the different irrational drug use behaviors mentioned are the following: males, lower educational groups, housewives, not having social insurance, continuous drug users.

**Conclusion:**

Irrational drug use behaviors are still observed in society. The fact that the risk is higher in lower education groups is an example of the negative consequences of limited health literacy. The fact that different risk groups have been identified for different behaviors related to irrational drug use shows that intervention studies on this subject should be directed to specific groups. The effect of having social security reveals its connection with universal health coverage and rational drug use.

## 1. Introduction

Rational drug use requires “patients to receive drugs appropriate to their clinical needs, in doses that meet their individual needs, for an adequate period, at the lowest cost to themselves and to society.”^1^ Rational drug use needs a systematic approach that involves diagnosis that is made correctly, giving the patient understandable information, and evaluation of the results of the treatment [1]. Consulting with healthcare professionals and patient compliance are indispensable for effective drug treatment [2].

Not prescribing drugs in accordance with treatment guidelines and using wrong and unnecessary drugs are characteristics of irrational drug use. Irrational drug use is one of the most basic health problems, which is a difficult habit to give up, all over the world, especially in developing countries. The World Health Organization states that more than half of all drugs are used inappropriately. Even if users receive treatments recommended by healthcare professionals using the services of healthcare organizations, it is their decision-making mechanisms that ultimately determine the drug use [3]. These decisions can be influenced by such factors as beliefs of family, friends, or society, information received from prescribing doctors and pharmacists who prepare/dispense the drug, and incentives acquired on the Internet [4]. Irrational drug use threatens patient safety. Negative consequences of drugs such as side effects, tolerance, resistance, and addiction can be seen in drug misuse and it also causes resources to be wasted [5].

The weight of noncommunicable diseases in the health workload is increasing. For instance, these diseases cause 71% of deaths worldwide^2^. The weight of noncommunicable diseases in the causes of mortality and morbidity is also among the reasons that make it necessary for rational drug use to continue to be on the agenda of countries’ health policies.

According to Ministry of Health data, there was an increase of 23.1% in the volume of pharmaceutical sales on a box basis from 2014 to 2019. In respect to daily antibiotic consumption per thousand people, Turkey ranks second among all OECD countries. On the other hand, in the comparison made within the scope of daily consumption of antihypertensive drugs, it is seen that Turkey ranks second from the last^3^. Drug use tends to increase in general, and accordingly, there are drug groups that are used more or less than the medical need makes rational drug use an important issue for Turkey.

In Turkey, the rate of self-medication is close to 50% in a population-based study and 90% in a study conducted in health institutions [6,7]. The rate of applying to the doctor to prescribe the drug was found to be 44% in a study conducted in primary care in Turkey [3]. The rate of drug use without reading the patient information leaflet (PIL) was found 39.1% in another study conducted in Turkey [8]. These local studies also show through different behaviors that irrational drug use is an important problem in Turkey.

The drug use behaviors of society should be investigated to prevent the negative effects of irrational drug use, which is affected by many factors. This study aims to evaluate the irrational drug use behaviors of individuals over the age of 18 who applied to a university hospital.

## 2. Materials and methods

### 2.1. Sampling

This sectional study was conducted on individuals over the age of 18 who applied to the outpatient clinics of Gazi University Health, Research, and Application Center in Ankara. While calculating the sampling size, the population of the study was taken as 16,650 (this is the number of individuals over the age of 18 who applied to the hospital in a week and the data were obtained from the statistics unit of the hospital), with 50% unknown frequency, 3% margin of error, and the design effect 1.0. The number of people to be included in the study was calculated as 1003, by calculating 25% lost data, it was aimed to reach 1254 people. Within the scope of this study, 1247 people were interviewed. The Open Epi program was used in the sampling calculation.

### 2.2. Data collection tools

A questionnaire on demographic information, socioeconomic variables, and drug use behaviors was applied to the participants by face-to-face interview technique. The questionnaire includes participants’ age, gender, education level, marital status, monthly household income, health insurance, perceived health level, chronic disease, and continuous drug use. There also were Likert-type questions that question the participants’ behaviors about irrational drug use. The questionnaire used included a total of 35 questions.

### 2.3. Application

Persons over the age of 18 who applied to Gazi University Health, Research and Application Center in Ankara were included in the study. Data collection was carried out with people who agreed to participate in the study between 20–25 September 2021 using the face-to-face interview method. Seventy-five intern doctors as interviewers and 6 research assistants as supervisors took part in the data collection phase.

Before starting the questionnaire, the participants were informed about the study, and verbal informed consent was obtained. People who did not want to participate in the study and who gave up participating in the study after starting the questionnaire were excluded from the study.

Gazi University Ethics Committee’s approval dated 07.09.2021 and numbered 2021-832 was received for the study.

### 2.4. Variables

The marital status information of the participants was collected as married, single, divorced, and widowed; the divorced and widowed were combined with the singles in the analysis and used as married and single.

The data of the people’s perceived health level were collected as very good, good, medium, bad, and very bad. Those who said very good and good were combined as good, and those who said very bad and bad were combined as bad in the analysis.

Most commonly used drugs without physician’s prescription and reasons for using the medication without physician’s suggestion were presented through the person using medication without a doctor’s prescription (n = 251). The questions mentioned here are the type of questions that can be answered with more than one choice.

Irrational drug uses investigated are “using medication without a doctor’s prescription”, “not using the drugs in the recommended dose and time”, “applying to the physician to prescribe the drug they want”, “keeping medication for later use at home”, “using drugs without reading the PIL inside the drug”, “using drugs without checking the expiration date”. The first two of the behaviors stated are five-point Likert questions, and the other behaviors are three-point Likert questions.

Likert scale questions were preferred in order to allow the creation of detailed cross-tables containing dependent variables with a 3-point or 5-point Likert scale. Different Likert types including 3-point or 5-point were chosen because it was thought that the most optimum answers could be obtained in terms of the scope of different questions. On the other hand, in this article, dependent variables were dichotomized to construct logistic regression models necessary to make a more concise and standardized assessment of irrational drug use-related behaviors.

When dichotomization was performed for 5-point Likert, those who sometimes, often, and always engage in the irrational drug use behavior under investigation were considered to be doing that behavior. When dichotomization was performed for 3-point Likert, those who sometimes and always engage in the irrational drug use behavior under investigation were considered to be doing that behavior.

### 2.5. Statistical analysis

Our independent variables are demographic and socioeconomic variables. Dependent variables are behaviors related to irrational drug use. Dependent variables are discrete variables so we used the chi-square test in bivariate analysis.

Logistic regression models were created for assessing factors related to irrational drug use. The independent variables with p < 0.25 in the bivariate analysis were included in the logistic regression model. Assumptions of logistic regression we considered are appropriate outcome types that include binary variables, independence of observations, and a sufficiently large sample size. While creating the regression models, “Enter” was used as “the variable selection method.” Type 1 error level was set as 0.05. Statistical analyses were performed using SPSS (version 23).

## 3. Results

A total of 1247 people were reached within the scope of the research. Some descriptive characteristics of the participants in the study are shown in Table 1. In terms of age variable, 39.5% of the participants are in the 45–64 age range, with a mean age of 43.81 ± 15.59 years and a median of 45 (min: 18–max: 86). Of the participants, 49.8% are women, 65.4% are married, 39.2% are college-university graduates. Of them, 41.9% work in an income-generating job, and 42.6% have a household income equivalent to expenses. Of the participants, 45% have at least one chronic disease, 44.8% use drugs continuously, 95.7% have health insurance, and 50.6% evaluate their health as good.

Table 2 shows the methods that the participants follow when they have a health problem. Considering the methods followed when there is a health problem, while 91.9% of the participants apply to the healthcare organization, 14.3% of them use home drugs.

Of the participants, 20.1% declared that they were using medication without a doctor’s prescription (self-medication) (n = 251). The three most commonly used drugs without a prescription are analgesics (94.0%), cold medications (52.6%), and stomach medications (32.7%). Not seeing the need to go to the doctor (79.3%) is one of the main reasons for using drugs without a doctor’s recommendation. Of the participants, 25.9% do not have time to go to the doctor, and 11.1% do not want to pay the examination fee, so they use drugs without a prescription (Table 2).

Table 3 and Table 4 show behaviors related to irrational drug use and the regression model of the factors affecting them. Of the participants, 20.1% used drugs without a prescription, 47.4% applied to the physician to prescribe the drug they wanted, 65% had drug at home for later use, 3.4% did not use the drugs at the recommended dose and time, 45.5% used the drug without reading the PIL within the pillbox, and 24.1% used the drug without looking at the expiration date. All of the logistic regression models were statistically significant.

Educational background, employment status, and perceived health level were found to be effective factors for drug use without a prescription (p < 0.05). High school graduates are more likely to use drugs without prescription, when they are compared to bachelors (OR = 1.659; 95% CI = 1.168–2.357). Compared to employees, retirees use fewer drugs without a prescription (OR = 0.522; 95% CI = 0.333–0.820). Those with bad perceived health use drugs without prescription more than those with good health (OR = 2.255; 95% CI = 1.320–3.851) (Table 3).

Continuous drug use and perceived health level were found to be effective factors for the behavior of applying to the physician to prescribe the drug they wanted (p < 0.05). Compared to those who do not use drugs, those who use them regularly (OR = 1.971; 95% CI = 1.325–2.932) and those who evaluate their health at a moderate level compared to those who evaluate their health at a good level (OR = 1.539; 95% CI = 1.167–2.029) applied to the physician more often to prescribe the drug they wanted (Table 3).

Household monthly income was found to be an effective factor for the presence of drugs for later use at home (p < 0.05). Those with a monthly income more than their expenses tend to store drugs more frequently for later use at home than those with less income than their expenses (OR = 1.454; 95% CI = 1.042–2.029).

Gender and health insurance were found to be effective factors for the behavior of not using drugs at the recommended dose and duration (p < 0.05). Men (OR = 2.347; 95% CI = 1.210–4.555) stop taking drugs more often before the recommended dose and duration or do not use them at all compared to women. Those who do not have health insurance stop using the drugs before the recommended dose and duration or do not use them at all more often than those with health insurance (OR = 3.945; 95% CI = 1.576–9.873)

Gender, marital status, educational status, employment status, and perceived health level were found to be effective factors for drug use behavior without reading the PIL in the pillbox (p < 0.05). Men compared to women (OR = 1.994; 95% CI = 1.495–2.660), and singles compared to married (OR = 1.687; 95% CI = 1.227–2.321) read the PIL less before using drugs. As the level of educational background decreases, the risk of using drugs without reading the PIL increases. Housewives compared to those who work (OR = 1.549; 95% CI = 1.016–2.361) use drugs more often without reading the PIL. Those with moderate perceived health use drugs less without reading the PIL than those with good perceived health (OR = 0.707; 95% CI = 0.533–0.939).

Gender, educational status, and perceived health level were found to be effective factors for drug use behavior regardless of the expiration date (p < 0.05). Men (OR = 1.880; 95% CI = 1.322–2.674) use drugs more frequently without looking at the expiration date compared to women. Those with moderate perceived health level use drugs less without looking at the expiration date compared to those with good (OR = 0.709; 95% CI = 0.508–0.989). As the level of educational background decreases, the risk of using drugs without looking at the expiration date increases.

## 4. Discussion

Sociodemographic characteristics of individuals affect many behaviors and decisions in daily life, as well as their drug use behaviors when a health problem occurs.

In this study, the vast majority of the participants stated that they applied to the healthcare organization when they had a health problem, and about one in seven people stated that they used the drugs they stored at home. Very few of the participants do nothing when they have a health problem. In a household-based study conducted in Manisa in 2013, similar to this study, it was found that three out of four of the participants applied to a healthcare organization when they had a health problem, one-fifth of them tried to self-medicate or be treated with the help of others, and very few of them did nothing [9]. The fact that our study was carried out on patients who applied to the hospital may be the reason for the higher frequency of applying to a healthcare organization. In a study conducted in Wuhan, China, in 2015, it was found that less than half of the individuals consult a doctor when they have a health problem, nearly half try to treat themselves, and about one-sixth of them do nothing [10]. This example illustrates the influence of different cultures on health behaviors.

The frequency of self-medication was found to be 20.1% in our study (using the medication without a doctor’s prescription). In a study conducted in İstanbul in 2019, the rate of self-medication was found to be 46.5%, and in a study conducted in Eskişehir in 2017, it was 90.6% [6,7]. In the study conducted in Eskişehir, while the rate of those with high school or higher education was 44.8, it was 75.7 in this study. As observed in the present study, as the education level increases, self-medication decreases generally, and this may be the reason for the different outcomes between the two geographically neighboring cities. On the other hand, considering that the study in Eskişehir included those who applied to a primary healthcare organization and our study included those who applied to a tertiary healthcare organization, it is noteworthy that there is a difference in terms of self-medication for different levels of healthcare organizations. In a 2014 study conducted in Brazil, the self-medication rate was found to be 16.1%, 62.9% in a study conducted with university students in Egypt, and 89.9% in a study conducted with nursing students in Turkey [11–13]. Differences in the health systems of countries and their access to health services also affect rational drug use behaviors. The differences between the results of our study and the results of other studies may be due to the differences between health systems and cultural differences of societies. Despite wide differences in outcomes, all studies identify self-medication as an important public health problem.

According to our study, the most commonly used drugs without a physician’s prescription is analgesic (94.0%). The most commonly used over-the-counter drug in our country and many countries is analgesics [9,13,14]. Pain is the most common symptom for which a patient seeks a drug, which is to be expected. [15]. Of course, applying to healthcare organizations even in the slightest pain can be a burden on the health system, especially in countries where the number of doctors is not sufficient, and using analgesics can reduce this burden. Nevertheless, it should not be forgotten that pain can be a symptom of other more serious diseases and making this distinction by a doctor is more appropriate for the principles of rational drug use.

In this study, it was found that the most common reason for self-medication was not the need to see a doctor (79.3%). Not being able to find time to go to the doctor and not wanting to pay the examination fee are among the other reasons. Not being able to find a doctor’s appointment is also among the reasons. In a study conducted in Brazil in 2017, it was determined that the most common reason for self-medication was the thought that the problem did not require a doctor’s appointment, with the second reason being the lack of time to go to the doctor [16]. In a study conducted with nursing students in Turkey in 2020, of the students, 53.2% reported the reason for self-medication as “not seeing it necessary to go to the doctor because the problem is insignificant” and 49.2% “having used the drug before” [13].

In this study, it was found that educational level is an effect on self-medication. In a study conducted in Sivas in 2020, in case of cold/flu, high school graduates were found to take more over-the-counter antibiotics than undergraduates [17]. The fact that employees use over-the-counter drugs more than retirees may be due to their inability to take time to apply to a health institution. The fact that those with bad perceived health use more over-the-counter drugs is an expected finding, given that these people will generally seek more drugs, and some of them will turn to over-the-counter drugs.

The rate of applying to the doctor to prescribe the drug they wanted was 47.4% in this study. This rate was found to be 44% for those who applied to family health centers in Ankara in 2013 [3]. Those who use drugs constantly apply to the doctor more to prescribe the drug they want than those who do not use drugs constantly. When patients apply to the doctor to have their prescription drugs prescribed, the demand for the drugs they want is a factor that increases this behavior and is an expected finding. In a study conducted with family doctors in Erzurum in 2016, it was determined that 83.8% of the family doctors prescribed the drugs that the patients wanted [18].

In the current study, 65% of the participants keep drugs for later use at home. In a study conducted to determine the rational drug use behaviors of individuals registered in a family health center in İstanbul in 2021, 76.8% of the participants stated that they have unfinished/unused drugs at home. In this study, 58.1% of the participants stated that they have an income above the minimum wage [8]. In a study conducted in the USA in 2018, it was determined that 79% of the households had at least one drug [19]. Its prevalence among university students in Egypt is 77.5% [12]. In this study, 58.1% of the participants stated that they have an income above the minimum wage [8]. It was also found in this study that those with higher incomes were more likely to have drugs at home, and that may be the reason why it is more likely in the USA.

In our study, 3.4% of the participants do not use the drugs in the appropriate dose and duration. In a study conducted in Ankara in 2004, this rate was found to be 28.6% [20]. In the study conducted in the family health center in İstanbul in 2021, the participants stated that 72.5% did not go to another doctor before the drugs they used were finished, 69.5% did not make any changes in the dose and duration of the drug, and 83.7% used the drug in accordance with the instructions [8]. In our study, the lowest frequency of irrational drug use behavior was found to be not using the drugs in the recommended dose and time. In other studies mentioned, it is observed that this frequency is partially lower than other irrational drug use behaviors. On the other hand, according to our study, the risk is higher in those who do not have social security. It is noteworthy that the odds ratio (OR) obtained for this variable is the highest value calculated for the variables.

In this study, the frequency of drug use without reading the PIL was found to be 45.5%. While this rate was found as 28.2% in a study conducted in İstanbul, this rate was found close to 39.1% in another study conducted in Ankara [3,8]. Educational background is an important factor affecting this behavior, and as the level of education increases, the rate of using drugs without reading the PIL decreases. Men use drugs without reading the PIL at a higher rate compared to women. Housewives also use drugs without reading the PIL at a higher rate than those who work. To promote rational drug use, training and studies should be carried out for housewives.

Regardless of the expiration date, the rate of drug use was found to be 24.1%. While this rate was stated as 21.5% in a study conducted in İstanbul, this rate was found to be 20.7% in a study conducted in Mersin [8,15]. In general, one out of every four-five people uses drugs without looking at the expiration date. In this situation, the use of expired drugs may result in ineffectiveness or delay in treatment or increase the likelihood of drug’s side effects. Expired drugs also cause economic losses.

One of the limitations of this study is that the results can only be generalized to the study group since the study was conducted on patients who were admitted to the hospital due to a health problem. Although it is estimated that it will not affect the results of the study, the fact that health professionals were not evaluated separately is another limitation.

Although the sale of over-the-counter drugs is prohibited by legal regulations, due to the ease of access to drugs in our country, drugs can be obtained from pharmacies without a prescription. As outlined in this study, individuals use drugs without going to the doctor because they do not want to pay the examination fee or cannot find the time. More studies are needed to detect irrational drug use behaviors in the community in various populations.

In conclusion, despite the efforts to generalize rational drug use, irrational drug use behaviors are still observed in society. Gender-differentiated patterns are seen for different behaviors associated with drug use. Men have an increased risk for all three of the behaviors studied. Low-educated groups have been identified as a risk group for not reading the PIL and not looking at the expiration date of drugs, which may be an example of the negative consequences of limited health literacy. The risk of not using the drugs in the recommended dose and time is higher in the group without social insurance. These results may be due to not being able to access the medicine even though it is needed. This point may be important in terms of universal health coverage. The fact that risk groups differ in drug-use related behaviors should be taken into account in intervention studies. Interventions that target specific risk groups for specific behaviors can bring more insight into the issues addressed in this paper.

## Figures and Tables

**Table 1:** Distribution of some descriptive characteristics of the participants.

	Number	(%)*
**Age groups (n = 1247)**		
24 years and under	180	14.4
25–44 years	440	35.3
45–64 years	492	39.5
65 years and older	135	10.8
**Gender (n = 1247)**		
Male	626	50.2
Female	621	49.8
**Marital status (n = 1247)**		
Married	816	65.4
Single	431	34.6
**Education Status (n = 1247)**		
Not a literate	18	1.4
Literate	21	1.7
Primary school graduate	264	21.2
High school graduate	455	36.5
College-university graduate	489	39.2
**Employment Status (n = 1247)**		
Employed	523	41.9
Housewife	268	21.5
Retired	218	17.5
Student	168	13.5
Unemployed	70	5.6
**Household monthly income (n = 1247)**		
Income less than expenses	364	29.2
Income equal to/equivalent to expenses	531	42.6
Income more than expenses	352	28.2
**Chronic disease status (n = 1247)**		
Yes	561	45.0
No	686	55.0
**Continuous drug use (n = 1247)**		
Uses	559	44.8
Does not use	688	55.2
**Health insurance (n = 1247)**		
Yes	1194	95.7
No	53	4.3
**Perceived health level (n = 1247)**		
Very good	155	12.4
Good	630	50.6
Intermediate	370	29.7
Bad	84	6.7
Very bad	8	0.6

* Column percentage

**Table 2:** The methods that the participants follow when they have a health problem, the drugs they use without a doctor’s prescription, and the reasons for using them without a doctor’s prescription.

The method people follow when they have a health problem (n=1247)*	Number	(%)
Applicants to the health institution	1147	91.9
Using home drugs	179	14.3
Using herbal/traditional method	133	10.7
Using medication in consultation with friends, acquaintancesneighbors, relatives	110	8.8
Taking medication in consultation with the pharmacist	99	7.9
Using drugs based on internet/social media resources	60	4.8
Does nothing	49	3.9
**Most commonly used drugs without physician’s prescription (n=251)** *		
Analgesic	236	94.0
Cold/flu drugs	132	52.6
Stomach medications	82	32.7
Vitamins/supplements	82	32.7
Muscle relaxants	72	28.7
Antibiotics	47	18.7
Aspirin/anticoagulants, oral	36	14.3
Allergy medications	35	13.9
**Reasons for using medication without physician’s suggestion (n=251)** *		
Not feeling the need to go to the doctor	199	79.3
Not having time to go to the doctor	65	25.9
Not wish to pay the examination fee	28	11.1
Other**	12	4.8

* Participants can give more than one answer.

** The other most common reason is not being able to find an appointment.

**Table 3:** Logistic regression models of the factors affecting self-medication, applying to a physician to prescribe the drug s/he wants and having drugs for later use at home.

	Using medication without a doctor’s prescription n (%)	OR (%95 CI)	Application to the physician to prescribe the drug s/he wants n (%)	OR (%95 CI)	Keeping medication for later use at home n (%)	OR (%95 CI)
**Gender**						
Male	131 (20.9)		263 (42.0)	1.00	381 (61.5)	1.00
Female	120 (19.3)		328 (52.8)	1.268 (0.962–1.671)	429 (69.6)	1.198 (0.900–1.593)
**Age groups**						
24 years and under	36 (20.0)		74 (41.1)	1.00	129 (72.9)	1.412 (0.707–2.820)
25–44 years	96 (21.8)		206 (46.8)	1.275 (0.783–2.077)	291 (66.9)	1.065 (0.647–1.751)
45–64 years	94 (19.1)		248 (50.4)	1.215 (0.702–2.105)	311 (63.5)	0.972 (0.631–1.498)
65 years and older	25 (18.5)		63 (46.7)	0.984 (0.489–1.978)	79 (59.0)	1.00
**Education status**						
Did not go to school	13 (33.3)	1.934 (0.858–4.355)	28 (71.8)	1.921 (0.836–4.412)	29 (74.4)	
Primary school graduate	45 (17.0)	1.014 (0.639–1.607)	131 (49.6)	0.847 (0.586–1.225)	161 (61.7)	
High school graduate	107 (23.5)	1.659 (1.168–2.357)*	216 (47.5)	1.102 (0.827–1.468)	307 (67.6))	
Bachelor and above	86 (17.6)	1.00	216 (44.2)	1.00	313 (64.9)	
**Household monthly income**						
My income is less than my expenses	72 (19.8)	1.00	190 (52.2)	1.00	232 (64.6)	1.00
My income is equal to/equivalent to my expenses	94 (17.7)	0.957 (0.671–1.363)	231 (43.5)	0.791 (0.596–1.050)	324 (61.4)	0.849 (0.637–1.133)
My income is more than my expenses	85 (24.1)	1.446 (0.988–2.116)	170 (48.3)	1.063 (0.773–1.461)	254 (72.8)	1.454 (1.042–2.029)*
**Employment status**						
Unemployed	13 (18.6)	0.809 (0.422–1.554)	32 (45.7)	0.953 (0.555–1.637)	44 (62.9)	0.935 (0.537–1.626)
Housewife	54 (20.1)	0.707 (0.462–1.082)	158 (59.0)	1.307 (0.869–1.964)	193 (72.6)	1.454 (0.979–2.159)
Retired	32 (14.7)	0.522 (0.333–0.820)*	97 (44.5)	0.932 (0.626–1.388)	120 (55.0)	0.751 (0.505–1.117)
Student	33 (19.6)	0.726 (0.455–1.159)	70 (41.7)	1.079 (0.640–1.819)	120 (72.7)	1.183 (0.689–2.033)
Employed	119 (22.8)	1.00	234 (44.7)	1.00	333 (64.4)	1.00
**Health insurance**						
Has health insurance	240 (20.1)		560 (46.9)	1.00	781 (66.0)	1.759 (0.984–3.143)
No health insurance	11 (20.8)		31 (58.5)	1.419 (0.771–2.611)	29 (54.7)	1.00
**Chronic disease status**						
Yes	121 (21.6)		295 (52.6)	0.771 (0.516–1.152)	361 (64.9)	
No	130 (19.0)		296 (43.1)	1.00	449 (66.0)	
**Continuous drug use**						
Uses	122 (21.8)	1.238 (0.900–1.704)	308 (55.1)	1.971 (1.325–2.932)*	364 (65.6)	
Does notuse	129 (18.8)	1.00	283 (41.1)	1.00	446 (65.5)	
**Perceived health level**						
Good	147 (18.7)	1.00	330 (42)	1.00	506 (65.3)	
Intermediate	73 (19.7)	1.143 (0.811–1.610)	209 (56.5)	1.539 (1.167–2.029)*	242 (65.6)	
Bad	31 (33.7)	2.255 (1.320–3.851)*	52(56.5)	1.287 (0.789–2.102)	62 (67.4)	
**In all participants (n = 1247)**	251 (20.1)		591 (47.4)		810 (65.0)	

* p < 0.05

**Table 4:** Logistic regression models of factors affecting some drug use behaviors.

	Not using the drugs in the recommended dose and time n (%)	OR (%95 CI)	Uses drugs without reading the informative text inside the drug n (%)	OR (%95 CI)	Uses drugs without checking the expiration date n (%)	OR (%95 CI)
**Gender**						
Male	30 (4.8)	2.347 (1.210–4.555)*	310 (49.5)	1.994 (1.495–2.660)*	169 (27.0)	1.880 (1.322–2.674)*
Female	13 (2.1)	1.00	258 (41.5)	1.00	132 (21.3)	1.00
**Age groups**						
24 years and under	9 (5.0)		88 (49.2)	1.542 (0.760–3.128)	44 (24.4)	
25–44 years	16 (3.6)		172 (39.1)	1.036 (0.606–1.771)	100 (22.7)	
45–64 years	15 (3.1)		244 (49.6)	1.430 (0.909–2.251)	122 (24.8)	
65 years and older	3 (2.2)		64 (47.4)	1.00		
**Marital status**						
Single	14 (3.3)		211 (49.1)	1.687 (1.227–2.321)*	112 (26.0)	
Married	29 (3.6)		357 (43.8)	1.00	189 (23.2)	
**Education status**						
Did not go to school	0 (0)		30 (76.9)	6.017 (2.551–14.19)*	23 (59.0)	7.937 (3.621–17.400)*
Primary school graduate	11 (4.2)		149 (56.4)	2.443 (1.692–3.529)*	84 (31.8)	2.806 (1.850–4.256)*
High school graduate	16 (3.5)		217 (47.7)	1.682 (1.256–2.251)*	120 (26.4)	2.115 (1.488–3.008)*
Bachelor and above	16 (3.3)		172 (35.2)	1.00	74 (15.1)	1.00
**Employment status**						
Unemployed	3 (4.3)		33 (471.1)	1.212 (0.693–2.121)	19 (27.1)	1.480 (0.806–2.718)
Housewife	6 (2.2)		140 (52.2)	1.549 (1.016–2.361)*	79 (29.5)	1.240 (0.763–2.016)
Retired	11 (5.0)		94 (43.1)	0.901 (0.602–1.350)	47 (21.6)	0.800 (0.529–1.209)
Student	5 (3.0)		78 (46.7)	0.681 (0.391–1.186)	38 (22.6)	0.888 (0.561–1.406)
Employed	18 (3.4)		223 (42.6)	1.00	118 (22.6)	1.00
**Household monthly income**						
My income is less than my expenses	17 (4.7)		159 (43.7)		85 (23.4)	
My income is equal to/equivalent to my expenses	17 (3.2)		243 (45.8)		124 (23.4)	
My income is more than my expenses	9 (2.6)		166 (47.2)		92 (26.1)	
**Health insurance**						
Has health insurance	37 (3.1)	1.00	540 (45.2)		280 (23.5)	1.00
No health insurance	6 (11.3)	3.945 (1.576–9.873)*	28 (52.8)		21 (39.6)	1.489 (0.798–2.779)
**Chronic disease status**						
Yes	17 (3.0)		275 (49.0)	1.015 (0.680–1.515)	146 (26.0)	0.834 (0.523–1.327)
No	26 (3.8)		293 (42.7)	1.00	155 (22.6)	1.00
**Continuous drug use**						
Uses	16 (2.9)		277 (49.6)	1.263 (0.848–1.880)	151 (27.0)	1.458 (0.918–2.315)
Does not use	27 (3.9)		291 (42.4)	1.00	150 (21.8)	1.00
**Perceived health level**						
Good	26 (3.3)		355 (45.3)	1.00	186 (23.7)	1.00
Intermediate	11 (3.0)		157 (42.4)	0.707 (0.533–0.939)*	81 (21.9)	0.709 (0.508–0.989)*
Bad	6 (6.5)		56 (60.9)	1.015 (0.615–1.674)	34 (37.0)	1.019 (0.600–1.733)
**In all participants (n = 1247)**	43 (3.4)		568 (45.5)		301 (24.1)	

* p < 0.05

## Footnotes

1 World Health Organization (2021). Promoting rational use of medicines [online]. Website https://www.who.int/activities/promoting-rational-use-of-medicines [accessed 30.08.2021]

2 World Health Organization (2021). Noncommunicable diseases [online]. Website https://www.who.int/health-topics/noncommunicable-diseases#tab=tab_1 [accessed 03.09.2021]

3 Turkish Ministry of Health (2021). Sağlık İstatistikleri Yıllığı [online]. Website https://sbsgm.saglik.gov.tr/Eklenti/40564/0/saglik-istatistikleri-yilligi-2019pdf.pdf [accessed 04.09.2021]
